# The Amyloid, Tau, and Neurodegeneration (A/T/N) Classification Applied to a Clinical Research Cohort with Long-Term Follow-Up

**DOI:** 10.3233/JAD-191227

**Published:** 2020-04-07

**Authors:** Gøril Rolfseng Grøntvedt, Camilla Lauridsen, Guro Berge, Linda R. White, Øyvind Salvesen, Geir Bråthen, Sigrid Botne Sando

**Affiliations:** a Department of Neurology and Clinical Neurophysiology, University Hospital of Trondheim, Trondheim, Norway; b Department of Neuromedicine and Movement Science, Faculty of Medicine and Health Sciences, Norwegian University of Science and Technology, Trondheim, Norway; c Unit for Applied Clinical Research, Faculty of Medicine and Health Sciences, Norwegian University of Science and Technology, Trondheim, Norway

**Keywords:** Alzheimer’s disease, amyloid, biomarkers, cerebrospinal fluid, classification, mild cognitive impairment, tau

## Abstract

**Background:**

The unbiased amyloid, tau, and neurodegeneration (A/T/N) classification is designed to characterize individuals in the Alzheimer continuum and is currently little explored in clinical cohorts.

**Objective:**

A retrospective comparison of the A/T/N classification system with the results of a two-year clinical study, with extended follow-up up to 10 years after inclusion.

**Methods:**

Patients (*n* = 102) clinically diagnosed as Alzheimer’s disease (AD) with dementia or amnestic mild cognitive impairment (MCI), and 61 cognitively healthy control individuals were included. Baseline cerebrospinal fluid core biomarkers for AD (Aβ_42_, phosphorylated tau, and total tau) were applied to the A/T/N classification using the final clinical diagnosis at extended follow-up as the gold standard.

**Results:**

A + T + N+ was a strong predictor for AD dementia, even among cognitively healthy individuals. Amnestic MCI was heterogenous, considering both clinical outcome and distribution within A/T/N. Some individuals with amnestic MCI progressed to clinical AD dementia within all four major A/T/N groups. The highest proportion of progression was among triple positive cases, but progression was also common in individuals with suspected non-Alzheimer pathophysiology (A-T + N+), and those with triple negative status. A-T-N- individuals who were cognitively healthy overwhelmingly remained cognitively intact over time, but in amnestic MCI the clinical outcome was heterogenous, including AD dementia, other dementias, and recovery.

**Conclusion:**

The A/T/N framework accentuates biomarkers over clinical status. However, when selecting individuals for research, a combination of the two may be necessary since the prognostic value of the A/T/N framework depends on clinical status.

## INTRODUCTION

Alzheimer’s disease (AD) is the most prevalent neurodegenerative disorder comprising 50–60% of dementia cases. With an aging population, the number of people with dementia worldwide is expected to quadruple by 2050 unless effective treatment or prevention becomes available [1]. It is now accepted that AD is a continuum and probably begins at least 15–20 years before symptom onset [2, 3]. During the last 15 years, research has moved increasingly toward the pre-dementia phase of AD, particularly mild cognitive impairment (MCI), during which there is a measurable loss of cognitive ability and some brain atrophy, but the patient is still capable of independent living. Criteria for MCI were presented in 2004 [4], and the type most often converting to AD is the amnestic form, where memory is affected. Detailed information on the progression process will be essential when effective treatment that hinders development of the disease becomes available. Society as a whole will also benefit since a 5-year delay in dementia onset has been estimated to reduce the number of dementia cases by 57% [5].

Clinical evaluation and cognitive test batteries alone frequently fail to provide the clinician with a precise diagnosis of AD [6]. The National Institute on Aging and Alzheimer’s Association (NIA-AA) Alzheimer’s Diagnostic Framework introduced a new, unbiased classification system in 2016 to apply validated biomarkers for the separation of AD from non-AD causes of impaired cognition. The classification uses three types of biomarkers to determine the extent of pathology typical of AD: A (amyloid, represented either by cerebrospinal fluid (CSF) levels of amyloid-β 42 (Aβ_42_) or amyloid plaque deposition in brain as seen with amyloid-PET); T (tau, measured as the level of CSF hyperphosphorylated tau (p-tau) or tangle-formation as seen by tau-PET); and N (neurodegeneration as shown by structural MRI, CSF levels of total tau (t-tau), or brain metabolism as measured with fluorodeoxyglucose (FDG-PET)). The framework thereby characterizes the AD spectrum by its biological presentation, and is independent of clinical assessment of cognitive status. It has been designated the A/T/N classification system and individuals can be classified as positive (+) or negative (–) for A, T and N, resulting in 8 possible A/T/N profiles [7].

The aim of this study was to explore the distribution of the A/T/N classification system in a clinical cohort and evaluate agreement with long term clinical outcome.

## MATERIALS AND METHODS

### Ethics

The present study was conducted according to the Helsinki Declaration. Written, informed consent was obtained from all patients or suitable proxies, and from all control individuals. The Trønderbrain Biobank has been licensed by the Norwegian Directorate for Health Affairs, and the research was approved by the Regional Committee for Medical Research Ethics (2010/226 REK Midt).

### Subjects

The demographic and associated data for the study cohort are shown in Table 1. A total of 102 patients were recruited through the Department of Neurology, University Hospital of Trondheim, by two neurologists with specialization in dementia disorders (SBS and GRG). Patients were included between 2009 and 2013 and were diagnosed as either having AD with dementia according to the NINCDS-ADRDA criteria [8], or amnestic MCI according to the International Working Group on Mild Cognitive Impairment Criteria [9]. Diagnosis was determined blind to biomarker concentrations in all cases.

**Table 1:** Demographic data of the clinical groups at baseline

Diagnosis	AD dementia	Amnestic MCI	Healthy individuals
N	38	64	61
Gender female (%)	20 (52.6)	34 (53.1)	40 (65.6)
Age (y)	63.5 (54–78)	64 (53–79)	68 (53–79)
*APOE* *ɛ*4 allele (%)	30 (78.9)	39 (60.9)	20 (32.8)
MMSE (max. 30)	23 (16–27)	28 (23–30)	30 (28–30)
TMTA (s)	60 (32–300)	51 (23–146)	43 (23–75)
TMTB (s)	161 (107–255)	109 (72–330)	94 (40–240)
CERAD TWT, delayed recall	1 (0–5)	3 (0–8)	7 (3–10)

AD, Alzheimer’s disease; MCI, mild cognitive impairment; TMTA, Trailmaking test A; TMTB, Trailmaking test B; TWT, Ten Word Test. Age, MMSE, TMTA, TMTB, and CERAD TWT delayed recall are given as median (range).

All patients were ethnic Norwegians between 54–78 years of age. General exclusion criteria were insufficient sight and hearing to complete the cognitive testing, a present psychiatric or malignant disease, use of anti-coagulating medication, or high alcohol consumption.

The control population consisted of 61 elderly volunteers between 57–84 years of age. They were recruited from societies for retired people in central Norway and assessed as being cognitively healthy for their age without signs of neurological disorders. Neurological examination, blood screening, and cognitive tests were performed on all participants, including the Mini-Mental State Examination (MMSE) [10], the CERAD Ten-Word Test (TWT) with delayed recall, as taken from ADAS Cog [11], Trailmaking test A and B [12], as well as cerebral 3T MRI at baseline and after two years. *APOE* genotyping was performed on blood samples according to the method described elsewhere [13].

Study participants were initially monitored over a period of two years. During this time, almost half of the patients with amnestic MCI at baseline progressed to AD dementia, as described elsewhere [14]. It was subsequently possible to carry out an extended follow-up of the cohort, with a median time of 9 years (range 6–10 years). The clinical diagnosis after the initial study period and at the extended follow-up was determined by the same neurologists, and was based on information obtained from clinical interviews, examinations, and medical records. The neurologists were blind to biomarker results. The same criteria for amnestic MCI and AD dementia were applied as at baseline. Frontotemporal dementia (FTD) and vascular dementia were determined according to criteria according to Brun et al. [15] and Roman et al. [16] respectively.

### Sampling and analysis of CSF

All participants underwent lumbar puncture at baseline. The procedure was usually carried out with the patient lying on their side, and CSF drawn at the L4/L5 intervertebral space. The pattern of CSF collection was the same for all study individuals, and usually performed early in the morning. The first 2.5 ml CSF was used for non-research purposes, including routine clinical investigation. Subsequent CSF samples (1 ml aliquots) were collected directly into 2 ml polypropylene cryovials (Corning) immersed in ice-water. Samples were kept in ice-water while being sent for storage, and frozen within 30 min of lumbar puncture unless centrifuged to remove erythrocyte contamination [17, 18]. Erythrocyte counts in the remaining samples were (mean±SD) 2.0±4.3 erythrocytes/*μ*l CSF, overall range 0–33 erythrocytes/*μ*l CSF, median 1 erythrocyte/*μ*l CSF. All samples were stored at –80°C until analysis, when they were slowly thawed in ice-water on the morning of analysis, and therefore underwent only this single freeze-thaw treatment.

CSF samples were analyzed in duplicate by commercially available enzyme-linked immunosorbent assay (ELISA) monoplex kits according to the manufacturers’ instructions from duplicate analyses. Biomarkers central to the A/T/N classification (Aβ_42_, t-tau, and p-tau) were analyzed by ELISA (Fujirebio Innogenetics) as described previously [19]. Coefficients of variation for the amyloid and tau markers have been given elsewhere [14, 20].

### Statistical analysis and grouping according to the A/T/N classification

Statistical analyses were carried out using SPSS version 25 (IBM). Normality was assessed through the inspection of QQ-plots, histograms, and the Shapiro-Wilks test of normality. CSF biomarkers had non-normal distributions, and group comparisons were carried out using the Kruskal-Wallis and Mann-Whitney U tests.

The healthy controls were significantly older than the patient groups at inclusion. The effect of age on analyte levels was examined by log-transforming the data to the natural logarithm to approximate a normal distribution, and applying a univariate linear model with age as co-variate. Since age was not found to affect the results presented here, it is not mentioned elsewhere in this article.

Assignment to A/T/N groups was based on cut-off levels of the core CSF biomarkers for AD, using Aβ_42_ for ‘A’, p-tau for ‘T’ and t-tau as ‘N’. The threshold levels were based retrospectively on the study material according to baseline CSF values in the AD dementia group compared to the healthy controls. After two-year follow-up none except one healthy control in these groups had changed diagnosis. Cut off values were calculated by maximizing Youden’s index [(sensitivity + specificity)-1] [21]. A cut-off value between controls and patients with early AD dementia for CSF Aβ_42_ from this material has previously been reported as 630 pg/ml [19]. Similarly, a cut-off level for CSF t-tau was calculated to be 394 pg/ml and for p-tau to be 66 pg/ml. Accordingly, CSF Aβ_42_ levels <630 pg/ml, t-tau levels >394 pg/ml, and p-tau levels >66 pg/ml were considered pathological.

## RESULTS

### Baseline biomarker levels in CSF

The concentration of baseline CSF biomarkers in the clinical groups at inclusion are presented in Table 2. The concentration of Aβ_42_ (“A”) was significantly lower in the AD group compared to the amnestic MCI group, and less than half the concentration in the healthy control group (both p < 0.01). It was also significantly lower in the amnestic MCI group compared to the controls (p < 0.01). The median Aβ_42_ concentrations in the AD group and the amnestic MCI group at baseline were both below the calculated threshold level for Aβ_42_ in this study (<630 pg/ml).

**Table 2:** Levels of biomarkers in CSF at baseline

Diagnosis	AD dementia	Amnestic MCI	Healthy individuals
CSF Aβ_42_ pg/ml	457 (212–1092)^a^	567 (173–1508)^a,b^	1013 (434–1674)
CSF t-tau pg/ml	612 (177–3162)^a^	418 (99–2325)^a,b^	287 (138–1314)
CSF p-tau pg/ml	81 (28–182)^a^	67 (16–169)^b^	54 (33–135)

AD, Alzheimer’s disease; MCI, mild cognitive impairment; CSF, cerebrospinal fluid. CSF biomarkers are given as median (range). ^a^Compared to healthy individuals, *p* < 0.01. ^b^Compared to AD dementia *p* < 0.01.

The concentration of p-tau (“T”) in CSF was significantly higher in the AD group compared to the amnestic MCI group and the controls (both p < 0.01). However, there was no significant difference in the CSF concentration of p-tau between the amnestic MCI and control groups. Nevertheless, median p-tau concentrations in both the AD and amnestic MCI groups at baseline were above the calculated threshold in this study (>66 pg/ml).

Similarly, the concentration of t-tau (“N”) in CSF was significantly higher in the AD group compared to the amnestic MCI group and controls (both p < 0.01), though the t-tau concentration was also significantly higher in the amnestic MCI group compared to controls (p < 0.01). Again, median t-tau concentrations in the AD and amnestic MCI groups at baseline were both above the calculated threshold level for this study (>394 pg/ml).

### A retrospective application of A/T/N to the clinical groups at baseline

**Fig.1:**
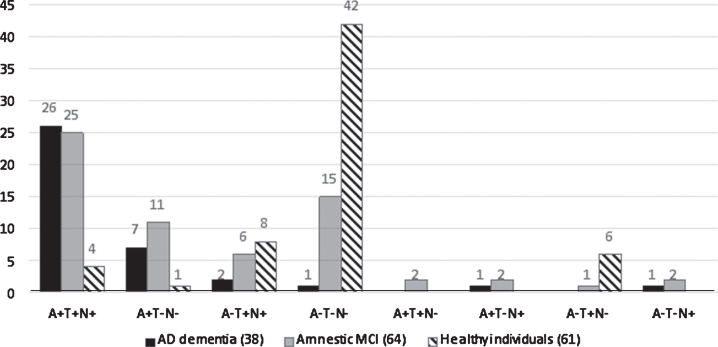
A retrospective application of A/T/N to the clinical groups at baseline. AD, Alzheimer’s disease; MCI, mild cognitive impairment. The figures over the histogram columns represent the number of individuals.

Figure 1 shows how individuals in the various clinical groups were distributed within the A/T/N classification system at baseline. Individuals classified as amyloid-positive (A+) were much more frequent in the patient groups: 89.9% in AD dementia (A + T + N + = 26, A + T-N- = 7, A + T-N + = 1) and 62.5% of those with amnestic MCI (A + T + N + = 25, A + T-N- = 11, A + T + N- = 2 and A + T-N + = 2), but only 8.2 % of controls (A + T + N + = 4 and A + T-N- = 1). Individuals classified as amyloid negative (A-) were most frequent in the control group (91.8%, A-T-N- = 42, A-T + N + = 8 and A-T + N- = 6), and 37.5% of those with amnestic MCI (A-T-N- = 15, A-T + N + = 6, A-T + N- = 1 and A-T-N + = 2), but only 10.5% of individuals AD dementia (A-T-N- = 1, A-T + N + = 2 and A-T-N + = 1).

### Sensitivity and specificity of the A/T/N classification of AD dementia at baseline compared to cognitively healthy control individuals at baseline

When a comparison was made between biomarker triple positive (A + T + N+) against biomarker triple negative (A-T-N-) in the affected (AD dementia) versus healthy (control) group, both sensitivity and specificity were over 90%. Individual assessment of the biomarkers showed best sensitivity and specificity for amyloid (A), around 90%. Neither T nor N were as sensitive or specific as amyloid. The data are shown in Table 3.

**Table 3:** Sensitivity and specificity of the A/T/N classification of AD dementia at baseline compared to cognitively healthy control individuals at baseline

	Sensitivity	Specificity
A	89.9 %	91.8 %
T	73.7 %	70.5 %
N	78.9 %	80.3 %

When all individuals in these two groups classified as A + T + N+ were compared to all those classified as A-T-N-, the sensitivity was 96.3 % and specificity was 91.3 %. Data were based on CSF levels of amyloid-β_42_ (A), phosphorylated tau (T), and total tau (N).

### Retrospective application of the A/T/N classification system to the AD group at baseline compared to clinical status after the extended follow-up period

The results of the A/T/N classification system, and clinical status after the extended follow-up period compared to baseline, are presented in Table 4. Only A + T + N+, A + T-N-, A-T + N+, and A-T-N- were included in the Table because of low numbers in other combinations (given in the Table legend). All patients diagnosed with AD dementia at baseline kept this diagnosis during a median follow-up of 8 years (range 6–10 years).

**Table 4:** A/T/N classification applied to clinical groups at baseline compared to clinical status in 2019

A/T/N classification	Diagnosis at inclusion 2009–2013			Diagnosis at follow-up in compared to inclusion		
	AD	aMCI	Controls	AD	aMCI	Controls
	*n* = 38	*n* = 64	*n* = 61	*n* = 38	*n* = 64	*n* = 61
A + T + N+	26	25	4	26 AD^*^	21 AD^**^	1 control
					2 aMCI	2 AD
					1 FTD	1 DBD
					1 DBD
A + T-N-	7	11	1	7 AD	5 AD	1 ALS
					3 aMCI
					1 epilepsy
					2 LTF
A-T + N+	2	6	8	2 AD	5 AD	3 controls
					1 aMCI	1 AD
						1 aMCI
						1 DBD
						2 LTF
A-T-N-	1	15	42	1 AD	3 AD	40 controls
					4 aMCI	2 LTF
					2 FTD^***^
					2 VaD^***^
					2 healthy
					1 DBD
					1 LTF

AD, Alzheimer’s disease dementia; aMCI, amnestic mild cognitive impairment; controls, cognitively healthy elderly control individuals; FTD, frontotemporal dementia; VaD, vascular dementia; DBD, died before diagnosis at two year follow up; LTF, lost to extended follow-up. Includes ^*^16 individuals; ^**^five individuals; ^***^one individual that died during extended follow-up. A/T/N was based on CSF concentrations of A = amyloid-β_42_, T = hyperphosphorylated tau, and N = total tau protein. The cut-off for amyloid-β_42_ was calculated as 630 pg/ml and levels lower than this were considered to be A + . The cut-off for hyperphosphorylated tau was 66 pg/ml and for total tau 394 pg/ml. Levels higher than these were considered respectively T + and N + . Combinations that were excluded due to low n were: A + T + N- (2 aMCI), A + T-N+ (1 AD, 2 aMCI), A-T + N- (1 aMCI, 6 controls) and A-T-N+ (1 AD, 2 aMCI).

Twenty-one of 25 amnestic MCI patients classified as A + T + N+ at baseline had progressed to AD dementia during the follow-up period, while only two remained stable. However, these two had been recruited late in the inclusion period and had a follow-up time of only 6 years. One patient with amnestic MCI classified as A + T + N+ had progressed clinically to FTD, and one had died before the two-year follow-up.

Of the 11 patients with amnestic MCI at baseline and subsequently classified as A + T-N-, five had progressed to AD dementia during the extended period, three remained stable, one was diagnosed with severe epilepsy, and two could not be traced.

Six patients with amnestic MCI at baseline were classified as A-T + N+. Of these, five progressed to AD dementia and one remained stable during extended follow-up.

Fifteen of the amnestic MCI patients at baseline were classified as A-T-N-. After extended follow-up, four of these patients had remained stable, three had progressed to AD dementia, four were diagnosed with FTD or vascular dementia, one died before the two-year follow-up, two were classified as being cognitively healthy, while the last could not be traced.

Of the 42 healthy controls at baseline that were classified as A-T-N-, 40 remained cognitively healthy and two were lost to follow-up over a median of 9 years (range 7–10). Eight controls were classified as A-T + N+ at baseline, of whom three remained cognitively healthy, one progressed to amnestic MCI and one to AD dementia, one had died before the two-year follow-up and two could not be traced. Of four classified as A + T + N+ at baseline, two progressed to AD dementia, one had died before the two year follow-up, and one remained cognitively healthy during extended follow-up. Only one control individual was classified as A + T-N-, and developed amyotrophic lateral sclerosis within a year of inclusion. No motor symptoms were present at baseline.

## DISCUSSION

In this study we used the clinical diagnosis after six to ten years of follow-up as the gold standard independent of biomarkers for AD dementia, amnestic MCI, and healthy controls, for a retrospective comparison with the A/T/N classification. CSF values of Aβ_42_ (“A” for amyloid), phosphorylated tau (“T” for tau), and total tau (“N” for neurodegeneration) were used [7]. The A/T/N system is recommended for research purposes only, but it is still of interest to see how it would be represented in a clinical cohort, and to explore its application in a diagnostic setting. The concept of the A/T/N system was only possible because numerous studies have identified typical changes in brain pathology and CSF during the development of AD. It is therefore unsurprising that most triple positive cases (A + T + N+) were found in the group of patients with AD dementia at baseline (where only four of 38 cases were amyloid negative), and in patients with amnestic MCI who converted to AD dementia during the initial study period or subsequent follow-up. Similarly, there was a large predominance of triple negative cases (A-T-N-) among cognitively-healthy control individuals.

The prevalence of suspected non-Alzheimer disease pathophysiology (SNAP) at baseline was relatively consistent across clinical groups (controls 13%, amnestic MCI 9%, and AD dementia 5%) and correlates well with other studies [22, 23]. These individuals had tau pathology (T+) and/or neurodegeneration (N+), but lacked amyloid deposition (A-), thereby distinguishing them from the typical biomarker profile of AD [24]. They often receive a clinical AD diagnosis, and in this study five of six individuals with amnestic MCI at baseline with A-T + N+ progressed to AD dementia. The average level of Aβ_42_ in the A-T + N+ (816 pg/ml) group was much higher than the threshold for Aβ_42_ in this study. Autopsy studies have shown poor correspondence between clinical and neuropathological diagnosis, and up to one third of patients diagnosed as clinical AD showed mixed pathologies for AD, Lewy body disease, and vascular disease. Such pathologies can therefore cause dementia and amnestic MCI that mimic AD, also in the absence of amyloid pathology [25–27]. It has also been suggested that in some patients changes in tau metabolism may precede amyloid accumulation in AD [28, 29], even though the amyloid response usually occurs first [30].

A related concept has been termed primary age-related tauopathy (PART). It is characterized by medial-temporal neurofibrillary pathology with few or no amyloid deposits, and is mostly seen among aged cognitively healthy individuals. PART can also be present in cognitively impaired individuals and is clinically diagnosed as AD in up to half the cases. Some claim PART is a pathologic entity distinct from AD, while others see it as a part of the AD spectrum [31, 32]. Our results are further support that pathological changes in amyloid metabolism are not requisite for the development of clinical AD.

The proportion of individuals with A-T-N- at baseline decreased with diagnostic severity, being most common among the controls. Indeed, none that could be traced had cognitive decline at extended follow-up, suggesting that the absence of all three biomarkers has good prognostic value for not developing dementia [33]. However, this only seems to be true for the controls in this study cohort. Individuals with amnestic MCI classified as A-T-N- at baseline had the most heterogenous clinical outcome in the longer term, including cases of FTD and vascular dementia, as well as remaining stable amnestic MCI or regaining healthy cognitive status. Twenty percent of those with amnestic MCI who were A-T-N- at baseline developed clinical AD dementia during extended follow-up. A study applying the A/T/N classification to a group of individuals in the Alzheimer’s Disease Neuroimaging Initiative (ADNI) cohort found that 8% with amnestic MCI who were classified as A-T-N- at baseline developed AD dementia after 36 months. This is a lower percentage than found in the present study, but the follow-up period was much shorter [34].

Individuals with amnestic MCI who were triple negative might have been expected to be a group containing most individuals with stable amnestic MCI or regaining healthy cognitive status after extended follow-up. This was not the case. Almost half of the A-T-N- individuals in this group developed some type of dementia during follow-up. Our results therefore suggest that A-T-N- has a different prognosis depending on whether the individual is cognitively healthy or has an amnestic MCI phenotype at baseline.

Taken together it is clear that the amnestic MCI group was by far the most diverse and interesting group in this study. Although they all had a similar phenotype at the outset, they were heterogenous in the longer term both as to how they sorted into the A/T/N classification, as well as their final clinical outcome within the classification groups.

The main advantage of this study was the possibility to carry out a long-term follow-up of a high proportion of study individuals in a cohort were clinical diagnoses were independent of biomarkers. Additionally, the same two neurologists evaluated all participants and carried out all diagnostic procedures. This improved the continuity of the study and only 4% were lost to follow-up. Since all participants were followed over such a long period it was not possible either in terms of manpower or finances to include more participants, which is perhaps the main limitation of this study. Classifying A/T/N with biomarkers determined in CSF is a highly cost-effective procedure since a single lumbar puncture is cheaper than expensive imaging techniques. However, implementing different imaging techniques included in the A/T/N framework might improve the accuracy of the classification and possibly increase its diagnostic sensitivity and specificity further in this study.

## Conclusion

The A/T/N system has the advantage of being unbiased and independent of clinical status and gives the possibility to study a clinical cohort by its pathophysiology. When selecting individuals for research, a combination of clinical evaluation and biomarkers may still be needed.
